# A Creative Solution for a Difficult Case: Endoluminal Negative Pressure Therapy for the Management of Colorectal Anastomotic Leak

**DOI:** 10.7759/cureus.93785

**Published:** 2025-10-03

**Authors:** Maziar Fazel Darbandi, Jessica Shanahan, David Hochman, Farhana Shariff

**Affiliations:** 1 Surgery, University of Manitoba, Winnipeg, CAN

**Keywords:** anastomotic leak, colorectal anastomotic leak, colorectal cancer, endoluminal negative pressure therapy, negative pressure wound therapy, presacral abscess

## Abstract

Rectal anastomotic leaks can lead to significant morbidity with potentially devastating consequences for patients’ intestinal continuity and quality of life, which are challenging to manage. With a growing interest in endoluminal negative pressure therapy (eNPT), which was first described for use in esophageal anastomotic leaks, the technique has been more recently adapted to colorectal anastomoses.

We discuss a case of a patient with rectal cancer, who underwent total neoadjuvant therapy, ultralow anterior resection, and diverting loop ileostomy. The patient suffered an anastomotic leak and presacral abscess. Attempts at conventional management to control pelvic sepsis were unsuccessful. We present the novel creation of an eNPT device (a manufactured one is not readily available in Canada), which allowed control of sepsis, substantial reduction in abscess size, and safe discharge home for the patient. This experience suggests this is a safe and feasible treatment alternative for this challenging problem.

## Introduction

Colorectal cancer (CRC) is the third most diagnosed cancer and the second highest cause of cancer-related deaths worldwide [1]. Rectal cancer has worse outcomes than more proximal colon cancers, as it accounts for about 45% of CRC-related deaths, even though it only accounts for about 30% of CRC diagnoses [2-4]. Management of rectal cancer is complex and requires a multidisciplinary approach and often a combination of surgery, systemic therapy, and radiation therapy [4].

Anastomotic leak is a major complication of colorectal surgery, with rates as high as 10-20% for coloanal anastomoses [5,6]. Contributing factors include increased surgical duration, steroid use, pre-operative radiation, anastomotic location, and renal failure [5,6]. The extent of dehiscence, along with the degree of morbidity, can vary widely, and reported management approaches range from non-surgical management with antibiotics to surgical interventions, including proximal diversion, pelvic drainage, stapling of the cavity, or complete resection of the anastomosis [5-10].

Endoscopic interventions for gastrointestinal anastomotic leaks are gaining popularity, with endoluminal vacuum therapy first described in 2008 by Weidenhagen et al. for colorectal anastomotic leaks [11]. In a follow-up to their case series, the authors quoted successful resolution of leaks in 97% of cases [6-10]. More recently, the use of endoluminal negative pressure therapy (eNPT) has been adapted for use in colorectal anastomoses, with systematic reviews consistently reporting clinical success rates of 85-88% [6,8-10]. Additionally, eNPT has been reported to allow patients to regain bowel continuity in the future, with Strangio et al. finding that patients could go on to have their ileostomy reversed in 55-92% of cases [12].

The eNPT approach appears to be relatively safe. The most significant complication is stricture, which is generally amenable to endoscopic dilatation [6,8-10]. Other complications include bleeding and re-accumulation of the abscess cavity [7,13]. Mortality has been reported in 0-12.5% of cases and has never been directly associated with the procedure itself, but rather due to the general clinical condition of the patient [10]. At present, there are no validated functional outcomes reported in the literature in patients with eNPT for colorectal anastomotic leak.

There are no reported strict indications or contraindications for the use of eNPT devices, although surgery is still the recommended intervention for hemodynamically unstable patients. Generally, patients are deemed appropriate for management of their colorectal anastomotic leak using an eNPT device if they are hemodynamically stable and have larger anastomotic defects (>2 cm) [7,9,11].

Manufactured eNPT apparatuses are not available in Canada; therefore, the use of this technique for the management of colorectal anastomotic leak has not become routine.

## Case presentation

A 61-year-old male underwent the RAPIDO protocol total neoadjuvant therapy for an ultralow, locally advanced T4bN2aM0 rectal cancer, with post-treatment staging pelvic MRI revealing excellent response to treatment. This was resected by low anterior resection with coloanal anastomosis and diverting loop ileostomy. Final pathology demonstrated a ypT3N0 lesion. His initial postoperative course was uncomplicated, with discharge one week postoperatively. On postoperative day 14, he presented to a peripheral hospital with dark blood per rectum, and a colonoscopy was pursued, where the patient reported severe pain on entry. A 30% dehiscence at the posterior aspect of the anastomosis was reported, and he unfortunately developed a presacral abscess measuring 7.5 x 7.6 cm, with resultant pelvic sepsis and high-output stoma. Despite intravenous and oral antibiotics, as well as operative transrectal drainage, he required prolonged and repeated admissions for persistent abscess and acute kidney injury. After discussing with colleagues and reviewing the literature, the application of an endoluminal vacuum therapy apparatus was deemed appropriate. As the patient was hemodynamically stable and had a large anastomotic defect (>2 cm), he was deemed appropriate for management using eNPT. The patient was continued on antibiotics over the duration of management with eNPT. Since this product is not available in Canada, an innovative solution was needed.

**Figure 1:**
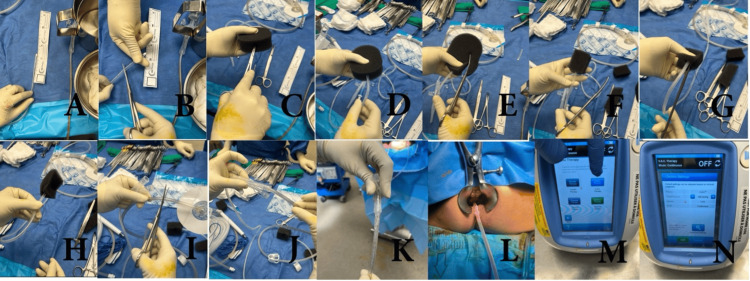
Crafting the endoluminal negative pressure therapy (eNPT) apparatus. (A, B) The nasogastric (NG) tube was measured and cut to size. (C, D) Tunnel in sponge and NG inserted. (E, F) Sponge cut to the cavity size. (G, H) Sutures secure the sponge to the NG tube. (I-K) NG tube connected to the vacuum-assisted closure (VAC) device tubing with an air-tight seal. (L) The device was inserted into the cavity under direct vision. (M, N) Device settings confirmed.

A negative pressure therapy (NPT) device was fashioned by first measuring the abscess cavity and cutting a black vacuum-assisted closure (VAC) sponge to the appropriate size. A 16 French nasogastric (NG) tube was then cut, ensuring that all side wall holes were within the sponge. A tunnel was created through the sponge, and the tip of the NG tube was inserted and secured with silk sutures through both structures to prevent sponge detachment and loss. The device was inserted into the abscess cavity under direct vision and connected to a VAC suction canister at an early pressure of -50 mmHg (Figure 1). Based on reported literature protocols, the patient went on to have NPT changes in the operating room every three to five days for a total of five changes with intermittent serial imaging. The pressure of the VAC suction canister during subsequent exchanges was increased to -100 mmHg after ensuring no early rectal pressure necrosis. Final on table flexible endoscopy confirmed excellent granulation of the pre-sacral cavity, with corresponding radiologic improvement. Furthermore, the pre-sacral cavity had mostly resolved, and the patient wished to go home after a long admission and time away from family. Therefore, it was felt that the patient could safely stop eNPT changes. The patient showed marked clinical improvement in ostomy output and was discharged home. Since discharge, follow-up imaging has demonstrated a small persistent presacral cavity; however, there is no active infection present, and no further hospitalizations have been required for this issue.

## Discussion

Anastomotic leak is a dreaded complication of gastrointestinal surgery [5]. A reoperation may be favored in the acute postoperative phase, especially in sick septic patients. Further out from surgery, the abdominal and pelvic cavities are too hostile, and the use of less invasive endoscopic techniques can be more favorable [13]. Endoscopic techniques include endoluminal vacuum devices, tissue sealants, and the over-the-scope-clip (OTSC) technique [13].

These have been proven to be safe and effective techniques, and, in a systematic review that included 75 patients, Chorti et al. showed that complete healing was achieved in 83.8% of patients using one or a combination of these endoscopic techniques [13].

There is scarce literature on quality-of-life measures for patients who suffer from anastomotic leak, especially those like our patient, who has not regained intestinal continuity. Ashburn et al. have published long-term functional and quality of life outcomes in patients who suffer from anastomotic leak compared to those who do not after restorative proctectomy for cancer [14]. Their study showed that at six months and one year after proctectomy, patients who had an anastomotic leak reported worse scores on physical and mental measures [14]. Three years after surgery, the worsened mental measures persisted [14]. Although the study does not exactly fit our patient scenario, it highlights the longstanding and chronic issues that patients who suffer from anastomotic leaks face; hence, timely and effective management is paramount.

With the growing body of evidence for the efficacy of using eNPT for the management of anastomotic dehiscence and leak, there is great potential for more routine use; however, eNPT devices are not readily available in Canada, forcing surgeons to creatively fashion safe alternatives. This report demonstrates that in a suitable center with appropriate expertise, this is a safe and effective solution for the management of complex rectal anastomotic leaks and resultant pelvic sepsis, in keeping with evidence from the use of manufactured devices [6-11]. As experience with this technique increases, work will be needed to explore functional outcomes in the future.

In accordance with the literature, we performed changes of the apparatus every three to five days, and the patient avoided complications from eNPT [10]. We were able to treat the patient adequately after five exchanges of the apparatus, but reviews have shown that up to 23 changes have been done [8,10]. This indicates that eNPT is effective in more chronic anastomotic leaks as well.

**Figure 2:**
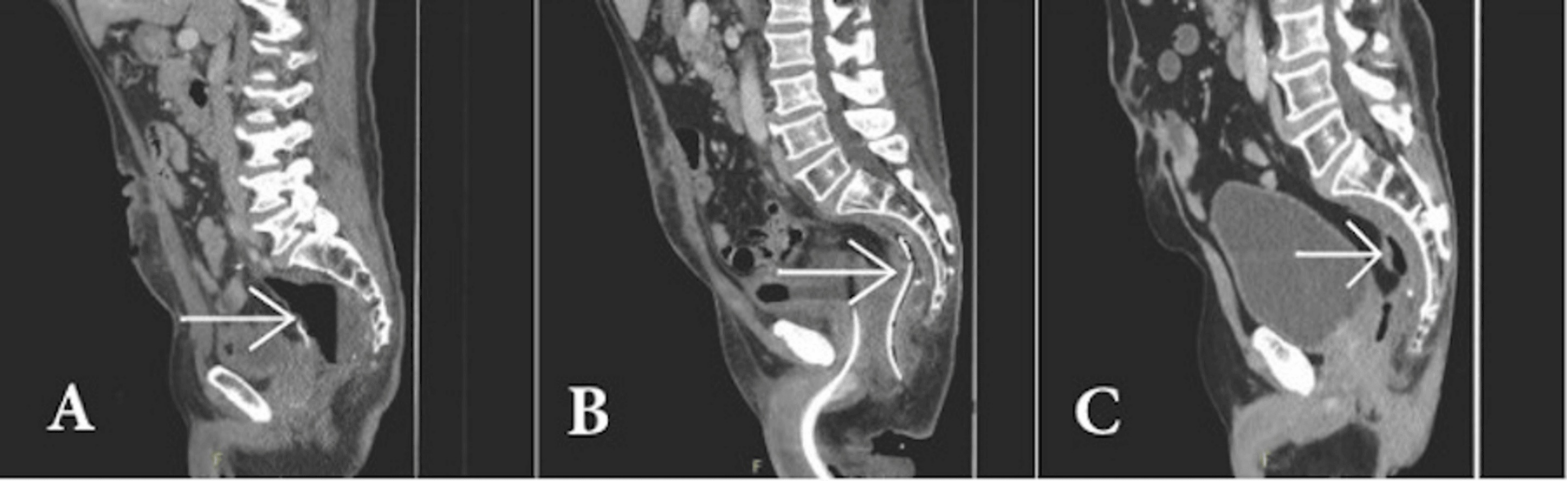
Size of the abscess cavity. (A) Post-anastomotic dehiscence with the arrow showing a large pelvic abscess. (B) An arrow showing the abscess cavity with a crafted endoluminal negative pressure therapy (eNPT) device inserted. (C) Six months post device removal, with the arrow showing residual cavity.

In this patient, the use of a fashioned eNPT device allowed for sepsis control and a significant reduction in the presacral abscess cavity, facilitating safe discharge without readmission. Figure 2 illustrates the improvement in the size of the patient's abscess cavity during and after application of the eNPT apparatus. The patient was offered additional eNPT changes to close the cavity completely and consider ileostomy reversal; however, due to already significantly prolonged time away from family, and good coping with the ileostomy, they chose to return home. The approach, however, achieved the goals of sepsis clearance, stoma output control, and, most importantly, allowed the patient to have their desired quality of life.

## Conclusions

Anastomotic leak after pelvic surgery is morbid, and while manufactured eNPT devices are not readily available outside of the USA, fashioning of similar devices is safe and effective at appropriate centers. As we share just one case in our report, this limits its generalizability but demonstrates the feasibility of this technique. The use of this apparatus in the correct clinical context allows for control of sepsis and facilitates safe discharge home.

## References

[1] Morgan E, Arnold M, Gini A (2023). Global burden of colorectal cancer in 2020 and 2040: incidence and mortality estimates from GLOBOCAN. Gut.

[2] Center MM, Jemal A, Smith RA, Ward E (2009). Worldwide variations in colorectal cancer. CA Cancer J Clin.

[3] Siegel RL, Fedewa SA, Anderson WF, Miller KD, Ma J, Rosenberg PS, Jemal A (2017). Colorectal cancer incidence patterns in the United States, 1974-2013. J Natl Cancer Inst.

[4] Schlechter BL (2022). Management of rectal cancer. Hematol Oncol Clin North Am.

[5] Ellis CT, Maykel JA (2021). Defining anastomotic leak and the clinical relevance of leaks. Clin Colon Rectal Surg.

[6] Dhindsa BS, Naga Y, Saghir SM (2021). Endo-sponge in management of anastomotic colorectal leaks: a systematic review and meta-analysis. Endosc Int Open.

[7] Mahendran B, Rossi B, Coleman M, Smolarek S (2020). The use of Endo-SPONGE® in rectal anastomotic leaks: a systematic review. Tech Coloproctol.

[8] Mussetto A, Arena R, Buzzi A, Fuccio L, Dari S, Brancaccio ML, Triossi O (2017). Long-term efficacy of vacuum-assisted therapy (Endo-SPONGE®) in large anastomotic leakages following anterior rectal resection. Ann Gastroenterol.

[9] Leeds SG, Mencio M, Ontiveros E, Ward MA (2019). Endoluminal vacuum therapy: how I do it. J Gastrointest Surg.

[10] Wikiel KJ, McConnell B, Bollinger D (2023). Endoluminal wound vacuum therapy for gastrointestinal leaks: current state and future directions. Ann Laparosc Endosc Surg.

[11] Weidenhagen R, Gruetzner KU, Wiecken T, Spelsberg F, Jauch KW (2008). Endoscopic vacuum-assisted closure of anastomotic leakage following anterior resection of the rectum: a new method. Surg Endosc.

[12] Strangio G, Zullo A, Ferrara EC (2015). Endo-sponge therapy for management of anastomotic leakages after colorectal surgery: a case series and review of literature. Dig Liver Dis.

[13] Chorti A, Stavrou G, Stelmach V, Tsaousi G, Michalopoulos A, Papavramidis TS, Kotzampassi K (2020). Endoscopic repair of anastomotic leakage after low anterior resection for rectal cancer: a systematic review. Asian J Endosc Surg.

[14] Ashburn JH, Stocchi L, Kiran RP, Dietz DW, Remzi FH (2013). Consequences of anastomotic leak after restorative proctectomy for cancer: effect on long-term function and quality of life. Dis Colon Rectum.

